# Uncoupling of Voltage- and Ligand-Induced Activation in HCN2 Channels by Glycine Inserts

**DOI:** 10.3389/fphys.2022.895324

**Published:** 2022-08-25

**Authors:** Sezin Yüksel, Michele Bonus, Tina Schwabe, Christopher Pfleger, Thomas Zimmer, Uta Enke, Inga Saß, Holger Gohlke, Klaus Benndorf, Jana Kusch

**Affiliations:** ^1^ Universitätsklinikum Jena, Institut für Physiologie II, Jena, Germany; ^2^ Institut für Pharmazeutische und Medizinische Chemie, Heinrich-Heine-Universität Düsseldorf, Düsseldorf, Germany; ^3^ John von Neumann Institute for Computing (NIC), Jülich Supercomputing Centre (JSC), Institute of Biological Information Processing (IBI-7: Structural Biochemistry) and Institute of Bio- and Geosciences (IBG-4: Bioinformatics), Forschungszentrum Jülich GmbH, Jülich, Germany

**Keywords:** HCN2 channels, voltage-dependent gating, cAMP-dependent gating, autoinhibition, patch-clamp technique, confocal patch-clamp fluorometry, molecular modeling, molecular dynamics simulations

## Abstract

Hyperpolarization-activated cyclic nucleotide-modulated (HCN) channels are tetramers that generate electrical rhythmicity in special brain neurons and cardiomyocytes. The channels are activated by membrane hyperpolarization. The binding of cAMP to the four available cyclic nucleotide-binding domains (CNBD) enhances channel activation. We analyzed in the present study the mechanism of how the effect of cAMP binding is transmitted to the pore domain. Our strategy was to uncouple the C-linker (CL) from the channel core by inserting one to five glycine residues between the S6 gate and the A′-helix (constructs 1G to 5G). We quantified in full-length HCN2 channels the resulting functional effects of the inserted glycines by current activation as well as the structural dynamics and statics using molecular dynamics simulations and Constraint Network Analysis. We show functionally that already in 1G the cAMP effect on activation is lost and that with the exception of 3G and 5G the concentration-activation relationships are shifted to depolarized voltages with respect to HCN2. The strongest effect was found for 4G. Accordingly, the activation kinetics were accelerated by all constructs, again with the strongest effect in 4G. The simulations reveal that the average residue mobility of the CL and CNBD domains is increased in all constructs and that the junction between the S6 and A′-helix is turned into a flexible hinge, resulting in a destabilized gate in all constructs. Moreover, for 3G and 4G, there is a stronger downward displacement of the CL-CNBD than in HCN2 and the other constructs, resulting in an increased kink angle between S6 and A′-helix, which in turn loosens contacts between the S4-helix and the CL. This is suggested to promote a downward movement of the S4-helix, similar to the effect of hyperpolarization. In addition, exclusively in 4G, the selectivity filter in the upper pore region and parts of the S4-helix are destabilized. The results provide new insights into the intricate activation of HCN2 channels.

## 1 Introduction

Hyperpolarization-activated cyclic nucleotide-modulated (HCN) channels are members of the superfamily of voltage-gated ion channels (Clapham, 1998). They are involved in a large variety of physiological and pathophysiological processes by playing a pivotal role in mediating electrical pacemaking activity of specialized cardiac and neuronal cells (Wahl-Schott and Biel, 2009). For mammalians, four isoforms have been described so far, HCN1-HCN4, all of them forming functional homo- or heterotetrameric channels (Biel et al., 2009).

**FIGURE 1:**
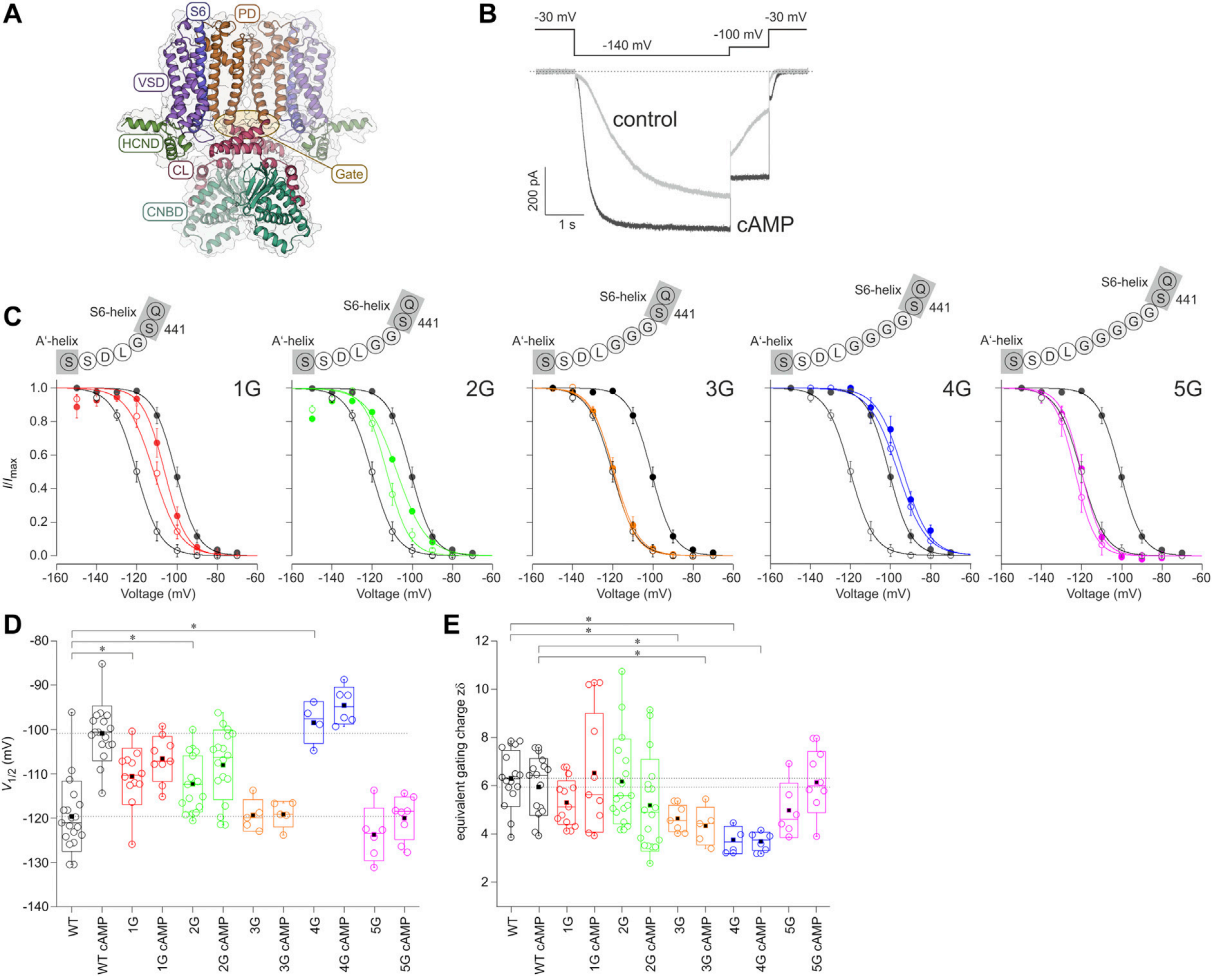
Effect of inserting different numbers of glycines between S6 and A′-helix on channel gating. **(A)** Structural model of the homotetrameric HCN2 channel (residues L136 to D650) as a side view. The gray bars depict the approximate location of the membrane bilayer. The voltage sensor domain (VSD), pore domain (PD), C-Linker (CL), cyclic nucleotide-binding domain (CNBD), and HCN domain (HCND) are illustrated. The arrow indicates the site of glycine insertion. **(B)** Exemplary current traces for HCN2 activation under control conditions without cAMP (grey trace) and with a saturating concentration of 10 µM cAMP (black trace). The used voltage protocol is shown above. **(C)** Steady-state activation relationships for all five mutated constructs in comparison to HCN2. Open symbols represent control conditions without cAMP, filled symbols represent recordings at 10 µM cAMP (*n* = 4–18). In all panels, black lines and symbols represent HCN2. The modified sequences are illustrated for each construct. **(D)** Box plots resenting *V*_1/2_ values without cAMP and with 10 µM cAMP with the following representations of the box features: center lines = medians, box limits = standard deviation, whiskers = minimum and maximum values, circles = individual recordings, squares = means. Horizontal black lines illustrate the wildtype values with and without cAMP for comparison. Significant differences are indicated by asterisks. **(E)** Box plot of *zδ* values. Box features are the same as in **(D)**. Significant differences are indicated by asterisks.

Each of the four subunits consists of four domains: 1) a transmembranal voltage sensor domain (VSD) including the helices S1 to S4, 2) a pore domain (PD), 3) an intracellular domain formed by the C-linker (CL) disk and the binding site for cyclic nucleotides (CNBD), and 4) an HCN domain at the channel’s periphery (HCND) (Figure 1A). The channel gate is formed by the intracellular ends of the S6-helices (Rothberg et al., 2002) that are arranged in a right-handed, tightly packed bundle at the inner entrance of the pore (Lee and MacKinnon, 2017). The four C-linkers form a gating-ring (Craven and Zagotta, 2004) between the transmembrane channel core and the ring-like structure built by the four CNBDs, as shown previously by resolving the crystal structure of isolated tetrameric CNBDs (Zagotta et al., 2003; Xu et al., 2010; Lolicato et al., 2011).

HCN channels are dually regulated by membrane hyperpolarization and cyclic nucleotide (cNMP) binding (Wang et al., 2001; Craven and Zagotta, 2006), with membrane voltage as the obligatory trigger and cNMP binding as a gating modulator. It has been proposed that the closed state of the channel is stabilized by a tight packing of the S4 to S6 helices and an unusually long S4-helix, protruding until the CL (Lee and MacKinnon, 2017). Membrane hyperpolarization would move the S4-helix into an intracellular direction, thereby disrupting the stabilizing effects of the mentioned components, presumably with a specific significance of only a few residues at the C-terminal part of S4 (Ramentol et al., 2020), leading to an opening of the gate. Additionally, previous work on HCN2 channels presented evidence for various interactions between the S4-S5 linker and the A′-helix of the CL during the gating process (Decher et al., 2004). It has been proposed that the gate opens by unwinding the S6-helix bundle as a consequence of an anti-clockwise rotation viewed from the extracellular side and an iris-like movement of the CL gating-ring (Craven and Zagotta, 2004; Shin et al., 2004; Lee and MacKinnon, 2017; Weissgraeber et al., 2017; Gross et al., 2018; Marchesi et al., 2018). In a recent study, Porro and co-workers assigned a regulatory role to the HCN-domain: it exerts an inhibitory effect on the voltage sensor via keeping it in a position unfavorable for channel opening (Porro et al., 2019). This supports the structural findings of Lee and MacKinnon, who suggested that the HCN domain stabilizes the closed state (Lee and MacKinnon, 2017).

According to the tetrameric structure of HCN channels, up to four cNMP molecules can bind to a channel. cNMP binding stabilizes a state that promotes the opening of the channels by relieving an autoinhibitory effect of the empty CNBD-CL portion on the gate (Wainger et al., 2001; Zhou and Siegelbaum, 2007; Craven et al., 2008). Consequently, both the rate and the extent of channel activation evoked by hyperpolarization are increased, and steady-state activation is shifted to less negative voltages (Robinson and Siegelbaum, 2003; Craven and Zagotta, 2006). Structural data showed that cNMP binding causes a similar rotation of the gating as VSD movement, but to a lesser degree and with a smaller resulting S6 displacement. Thus, cAMP supports activation by initiating the rotation of the gate-forming helices towards opening (Lee and MacKinnon, 2017).

In the present study, we focused on the mechanism of how cAMP binding controls the channel gate in the PD. Our strategy was to uncouple the CL from the S6 gate by inserting one to five glycine residues between the S6 and the A′-helix and to identify the resulting functional effects on both the activation gating and, reciprocally, on cAMP binding. We furthermore probed the effects of the additional amino acid sequences (AAASs) between channel gate and C-linker on the structural dynamics and statics of full-length HCN2 by molecular dynamics simulations and Constraint Network Analysis (CNA).

Here We show that the average residue mobility of the CL and CNBD domains is increased in 1G through 5G, which results in a destabilized S6 gate in all constructs. A stronger downward displacement was identified for 3G and 4G, which was not observed in HCN2 and the other constructs. This downward displacement loosens the contacts between the S4-helix and the CL, promoting a downward movement of the S4-helix similar to the effect of hyperpolarization. Exclusively in 4G, two additional effects distant from the CL-CNBD were observed: the selectivity filter in the upper pore region and parts of the S4-helix are destabilized. These computational interpretations can explain the lost cAMP effect by inserting only a single glycine, the observed shifts of steady-state activation to depolarized voltages, and the accelerated activation time courses by all constructs, as well as the exceedingly large effects observed for 4G. Our results help to improve our understanding of the activation mechanism in HCN2 channels.

## 2 Materials and Methods

### 2.1 *Xenopus laevis* Oocytes as Heterologous Expression System

Oocytes were surgically removed from adult female South African claw frogs *Xenopus laevis* under anesthesia with 0.3% tricaine methanesulfonate (MS-222) (Pharmaq Ltd. Fordingbridge, United Kingdom). After removal, the oocytes were treated with collagenase A (3 mg/ml; Roche, Grenzach-Wyhlen, Germany) for 105 min in Ca^2+^-free Barth’s solution containing (in mM) 82.5 NaCl, 2 KCl, 1 MgCl_2_, and 5 Hepes, pH 7.5. Oocytes of stages IV and V were manually dissected and injected with cRNA encoding either mHCN2 channels of Mus musculus or the mHCN2 mutants 1G, 2G, 3G, 4G, 5G, respectively. After injection with cRNA, the oocytes were incubated at 18°C for 2–6 days in Barth’s solution containing (in mM) 84 NaCl, 1 KCl, 2.4 NaHCO_3_, 0.82 MgSO_4_, 0.41 CaCl_2_, 0.33 Ca(NO_3_)_2_, 7.5 TRIS, pH 7.4. Oocytes harvested in our lab were complemented with ready-to-use oocytes purchased from Ecocyte Bioscience (Dortmund, Germany). The surgery procedures were carried out in accordance with the German Animal Welfare Act with the approval of the Thuringian State Office for Consumer Protection on 30.08.2013 and 09.05.2018.

### 2.2 Molecular Biology

One to five additional glycines were introduced into the mouse HCN2 cDNA (UniProt ID O88703 including two modifications, G8E and E55G without functional relevance) between residues S441 and L442 in mouse pGEM-HCN2 (Kusch et al., 2010) using overlapping PCR. First, PCRs were set up using forward outside primer (5′-CCT​GCT​GGG​ATC​CGA​ATT​CCA​CCA​TGG​ATG​CGC​G-3′) and reverse primer introducing the glycine (1G: 5′-GTG​ACG​AAT​CCA​GGC​CGG​ACT​GGA​TGA​GC-3'; 2G: 5′-GTG​ACG​AAT​CCA​GGC​CGC​CGG​ACT​GGA​TGA​GC-3'; 3G: 5′-GTG​ACG​AAT​CCA​GGC​CTC​CGC​CGG​ACT​GGA​TGA​GC-3'; 4G: 5′-CGT​GAC​GAA​TCC​AGT​CCG​CCT​CCG​CCG​GAC​TGG​ATG-3′) as well as reverse outside primer (5′-CTC​GTG​AGC​AAG​CAG​ATC​TCC​CCG​AAA​TAG​GAG​C-3′) and forward primer introducing the mutation (1G: 5′-CTC​ATC​CAG​TCC​GGC​CTG​GAT​TCG​TCA​C-3'; 2G: 5′-GCT​CAT​CCA​GTC​CGG​CGG​CCT​GGA​TTC​GTC​AC-3'; 3G: 5′-GCT​CAT​CCA​GTC​CGG​CGG​AGG​CCT​GGA​TTC​GTC​AC-3'; 4G: 5′-CTG​CGC​TCA​TCC​AGT​CCG​GCG​GAG​GCG​GAC​TGG​ATT​C-3′). The PCR products were used as templates in a final PCR using the two outer primers containing restriction sites for EcoRI and BglII, respectively. The resulting fragments were subcloned into the pGEM-HCN2. To construct 5G we used pGem-HCN2-4G as a template and the following internal primer pair: 5′-CCG​GCG​GAG​GCG​GGG​GAC​TGG​ATT​CGT​CAC​GGC​G-3′ and 5′-TGA​CGA​ATC​CAG​TCC​CCC​GCC​TCC​GCC​GGA​CTG​GAT​GAG-3’. The flanking forward and reverse primers were: 5′-CTCCCTGCGGATGTTCGGCA-3′and 5′-ATT​CCT​CCA​GCA​CCT​CGT​TGA-3′, respectively. The recombinant PCR product was inserted as a PsyI/BglII fragment into the corresponding sites of pGEM-HCN2. A thermostable DNA polymerase with proofreading activity was used for the respective PCR reactions (Pfu DNA polymerase, Promega, Madison, United States). The accuracy of the sequences of the inserts was confirmed by restriction digests and sequencing (Microsynth, Balgach, Switzerland). cRNAs were prepared using the mMESSAGE mMACHINE T7 Kit (Ambion).

### 2.3 Electrophysiological Experiments

Macroscopic currents were recorded using the inside-out configuration of the patch-clamp technique. All measurements were started after a delay of 3.5 min to minimize run-down phenomena. Patch pipettes were pulled from quartz tubings with outer and inner diameters of 1.0 and 0.7 mm (VITROCOM, New Jersey, United States), respectively, using a laser puller (P-2000, Sutter Instrument, Novato, United States). The pipette resistance was 1.2–2.1 MOhm. The bath solution contained (in mM) 100 KCl, 10 EGTA, and 10 Hepes, pH 7.2, and the pipette solution contained (in mM) 120 KCl, 10 Hepes, and 1.0 CaCl2, pH 7.2. For parts of the experiments, a saturating concentration of 10 µM cAMP (BIOLOG LSI GmbH & Co. KG, Bremen, Germany) was applied with the bath solution. A HEKA EPC 10 USB amplifier (Harvard Apparatus, Holliston, United States) was used for current recording. Pulsing and data recording were controlled by the Patchmaster software (Harvard Apparatus, Holliston, United States). The sampling rate was 5 kHz. The holding potential was generally −30 mV. Maximally two membrane patches were excised from one individual oocyte. For steady-state activation curves, relative current values for each recording were fitted individually (see Quantification and statistical analysis).

### 2.4 Quantification and Statistical Analysis

Steady-state activation curves were analyzed by fitting the Boltzmann equation to each individual recording using the OriginPro 9.0G software (Northampton, United States):

$$\frac{I}{I_{max}}=\frac{\frac{I}{I_{max,satV}}}{1+e^{\frac{z\delta F\left(V-V_{1/2}\right)}{RT}}}$$ (1)

*I/I*_max_ is the relative current, *I/I*_max, satV_ is the relative current at a saturating voltage and the actual cAMP concentration, *V*_1/2_ is the voltage of half-maximum activation, and *zδ* the effective gating charge. *F*, *R*, and *T* are the Faraday constant, the molar gas constant, and the temperature in Kelvin, respectively. The time courses of current activation were fitted with a single exponential starting after an initial delay using the OriginPro 9.0G software (Northampton, United States):

$$I\left(t\right)=Ae^{\frac{-t}{\tau }}$$ (2)

*A* is the amplitude, *t* the time, and *τ* the time constant for activation.

Experimental data are given as mean ± standard error of the mean (SEM). Statistical analysis was performed by an unpaired Student’s t-test. A value of *p* < 0.05 was accepted as statistically significant.

### 2.5 Confocal Patch-Clamp Fluorometry

The fluorescence intensity in the patch quantifying ligand binding was measured by patch-clamp fluorometry (Zheng and Zagotta, 2000; Zheng and Zagotta, 2003) combined with confocal microscopy (Biskup et al., 2007; Kusch et al., 2010). As fluorescent ligand, we used 8-AHT-Cy3B-cAMP (f1cAMP), a cAMP derivative in which the fluorescent dye Cy3B (GE Healthcare, Frankfurt, Germany) was linked via an aminohexylthio spacer to position 8 of the adenosine moiety (Otte et al., 2018; Otte et al., 2019). The recordings were performed with an LSM 710 confocal microscope (Carl Zeiss AG, Jena, Germany). They were triggered by the ISO3 software (MFK, Niedernhausen, Germany). To distinguish the fluorescence of the non-bound f1cAMP from that of the bound f1cAMP, a second, chemically related dye, DY647 (Dyomics GmbH, Jena, Germany), was added to the bath solution. The 543 and 633 nm lines of a He-Ne laser were used to excite f1cAMP and DY647, respectively. For quantifying the bound fcAMP, the fluorescence intensities of the red and the green channels were corrected for small offsets, and the fluorescence in the red channel was scaled to the fluorescence in the green channel in the bath. The difference between the measured green and the scaled red profile for each pixel of the confocal image represents the fraction of the fluorescence signal originating from the bound f1cAMP. Only the free patch membrane (patch dome) was used to quantify binding by setting a mask at a region of interest. The fluorescence, *F*, was averaged over all pixels inside this mask and normalized in each patch with respect to the fluorescence at saturating [f1cAMP] and full channel activation (−130 mV), *F*_max_. The recording rate of the confocal images was 10 Hz.

Concentration-binding relationships were analyzed by fitting the Hill equation to the mean data using the OriginPro 9.0G software (Northampton, United States):

$$\frac{F}{F_{max}}=\frac{1}{1+{\left(\frac{BC_{50}}{x}\right)}^{H_{b}}}$$ (3)

with *F* being the actual fluorescence intensity, *F*_max_ the maximal current amplitude at a saturating f1cAMP concentration and −130 mV, *BC*_50_ the concentration of half-maximum binding, and *H*_b_ the Hill coefficient.

### 2.6 Computational Studies

To determine if changes in the structural dynamics of specific regions in the 1G-5G constructs can be related to the electrophysiological results, we applied a combination of comparative modeling, molecular dynamics (MD) simulations, and rigidity analyses.

#### 2.6.1 Comparative Structural Modeling of mHCN2 Wildtype and the mHCN2-1G, -3G, -4G, and -5G Constructs

We generated structural models of the murine HCN2 wildtype channel (mHCN2; UniProt ID: O88703) and the corresponding 1G, 3G, 4G, and 5G constructs (Ser441_Leu442insGly_1-5_) using the RosettaCM method (Song et al., 2013) within the Rosetta macromolecular modeling, docking and design software (Koehler Leman et al., 2020). We did not generate a structure for 2G, given that the electrophysiological phenotype of 2G was similar to 1G with respect to *V*_1/2_. We selected two HCN1 structures (PDB IDs: 5U6O (Lee and MacKinnon, 2017) and 6UQG (Lee and MacKinnon, 2019)) and one HCN4 structure (PDB ID: 6GYN (Shintre et al., 2019)) as template structures for the hybridization approach in RosettaCM (Song et al., 2013). Among the available template structures with the highest sequence identity to HCN2, these structures also showed the highest structural similarity to each other. By excluding template structures in other conformational states, we aimed to avoid hybridization artifacts that might result from erroneous recombination of template structures in different conformational states. To predict the secondary structure of the protein for 3mer- and 9mer-fragment picking, we used both the server implementation of PSIPRED (Buchan et al., 2013; Buchan and Jones, 2019) and the corresponding standalone version (Jones, 1999), as well as the RaptorX-Property server (Wang et al., 2016). We used the standalone version of PSIPRED with a position-specific scoring matrix obtained from a PSI-BLAST (Altschul et al., 1997) run against the NCBI NR database (as of 05/2020) executed with the same arguments as specified in the Rosetta internal utility make_fragments.pl. To identify residues in transmembrane regions for the RosettaMP framework (Alford et al., 2015), we submitted the threaded template structures to the PPM Web Server (Lomize et al., 2012) and considered the consensus transmembrane residues in all structures in the final span file. We determined the required centroid and full atom parameter sets for cAMP using the Rosetta tool molfile_to_params.py with the cAMP conformers in 6UQG and 6UQF (Lee and MacKinnon, 2019). Subsequently, using equal weights for all template structures, we generated 100 symmetric models for the wildtype channel and each glycine linker construct. Then, maintaining symmetry, we relaxed each lowest-energy model using eight repetitions of the FastRelax Mover in RosettaScripts (Fleishman et al., 2011) with the franklin2019 scoring function (Alford et al., 2020), again creating 100 models. We considered the relaxed structure with the lowest total energy as the final model for all subsequent steps.

#### 2.6.2 MD Simulations

##### 2.6.2.1 System Setup

The structures of wildtype mHCN2 and the mHCN2-1G, -3G, -4G, and -5G constructs were embedded in a membrane bilayer consisting of approximately 34% cholesterol, 36% 1-palmitoyl-2-oleoyl-*sn*-glycero-3-phosphocholine (POPC), 17% 1-palmitoyl-2-oleoyl-*sn*-glycero-3-phosphoethanolamine, and 13% 1-palmitoyl-2-oleoyl-*sn*-glycero-3-phospho-l-serine (composition adjusted from ref. (Casares et al., 2019) to the lipids available in the force field) using CHARMM-GUI (Jo et al., 2008; Wu et al., 2014) and solvated with the OPC water model (Izadi et al., 2014) such that the minimum thickness of the water slab on top of and below the protein/membrane system was 25 Å. KCl was added to the system such that the charges of protein and membrane were counterbalanced and that its total concentration was ∼150 mM. Parameters for the protein and lipids were taken from the ff19SB force field (Tian et al., 2020) and the lipid17 force field (Gould et al., 2018), respectively; cAMP was described with parameters from the GAFF2 force field (Wang et al., 2004; Vassetti et al., 2019) and electrostatic point charges derived from a multiconformational RESP fit (Besler et al., 1990; Bayly et al., 1993; Wang et al., 2000).

##### 2.6.2.2 Simulation Protocol

MD simulations were performed using the mixed-precision (SPFP) GPU implementation (Le Grand et al., 2013) in the Amber 20 package (Case et al., 2020). Unless specified otherwise, a time step of 4 fs was defined for integration using a topology file with repartitioned hydrogen masses. The Langevin thermostat (Pastor et al., 1988; Loncharich et al., 1992) with a collision frequency of *γ* = 1.0 ps^−1^ and a target temperature of *T* = 300 K was used for temperature control. Covalent bonds to hydrogen atoms were constrained using the SHAKE algorithm (Ryckaert et al., 1977) with a tolerance of 10^–5^ Å. The Particle Mesh Ewald method (Darden et al., 1993) was used to compute long-range electrostatic interactions; short-range electrostatic and van der Waals interactions were computed with a cutoff of 10 Å.

To mitigate unfavorable contacts of the water molecules and the lipid tails in the initial simulation systems, these structural elements were first minimized for 2,500 steps using the steepest descent algorithm, followed by 2,500 steps of minimization with the conjugate gradient algorithm. Harmonic positional restraints with force constants of 2.5–10.0 kcal mol^−1^ Å^−2^ were applied to the remaining structural elements (Table 1). Retaining the restraints and starting at *T* = 100 K, the system was then thermalized for 50.0 ps in the NVT ensemble to reach the target temperature; this initial simulation step was performed with an integration time step of 1 fs to ensure a stable integration even at high system energies. The timestep was gradually increased to 4 fs in the next seven equilibration steps (total simulation time: 950 ps) performed in the NPT ensemble with semiisotropic pressure scaling. In parallel, the restraints were gradually removed (Table 1). The resulting system was used for five production runs of 1 μs length each, giving a total length of 30 μs for all production runs.

**TABLE 1:** Simulation scheme. Characteristics of the minimization, thermalization, equilibration, and production steps in the MD simulations of the HCN2 systems and the 1G-5G constructs.

Process	Number of Steps	Algorithm	Restrained Structures and Force Constant [kcal mol^−1^ Å^−2^]					
			*PBB* ^a^	*PSC* ^b^	*ChO* ^c^	*LiP* ^d^	*Ion* ^e^	*CMP* ^f^
Minimization	2,500/2,500	Steepest descent/Conjugate gradient	10.0	5.0	2.5	2.5	10.0	2.5

Process	Simulation Time [ps]	Ensemble/Time Step [fs]	Restrained Structures and Force Constant [kcal mol^−1^ Å^−2^]					
			*PBB* ^a^	*PSC* ^b^	*ChO* ^c^	*LiP* ^d^	*Ion* ^e^	*CMP* ^f^
Thermalization	50.0	NVT/1.0	10.0	5.0	2.5	2.5	10.0	2.5
Equilibration 1	100.0	NPT/2.0	5.0	2.5	2.5	2.5		2.5
Equilibration 2	100.0	NPT/2.0	2.5	1.0	1.0	1.0		1.0
Equilibration 3	100.0	NPT/4.0	1.0	0.5	0.5	0.5		0.5
Equilibration 4	100.0	NPT/4.0	0.1	0.1	0.1	0.1		0.1
Equilibration 5	100.0	NPT/4.0	0.1	-	-	-	-	-
Equilibration 6	150.0	NPT/4.0	0.05	-	-	-	-	-
Equilibration 7	300.0	NPT/4.0	-	-	-	-	-	-
Production	10^6^	NPT/4.0	-	-	-	-	-	-

a protein backbone.

b protein side-chain heavy atoms.

c cholesterol oxygen atoms.

d lipid phosphorus atoms.

e ions.

f cAMP.

#### 2.6.3 Rigidity Analysis

Rigidity analysis was performed with the CNA software package (Pfleger et al., 2013b). CNA efficiently decomposes a constraint network into rigid clusters and interconnecting flexible hinge regions by applying rigidity theory (Hermans et al., 2017). Whether a region in a biomolecule is flexible or rigid may depend on remote structural details, which makes rigidity analysis an attractive tool for studying altered structural stability due to distant influences (Jacobs and Hendrickson, 1997; Moukarzel and Duxbury, 1999).

Networks of covalent and non-covalent interactions (hydrogen bonds including salt bridges and hydrophobic tethers) were constructed from conformational ensembles extracted from MD trajectories of HCN2 and the HCN2-1G-5G constructs using the FIRST software (v.6.2) (Jacobs et al., 2001), for which CNA is a front and back end. The strength of the hydrogen bonds (including salt bridges) was assigned via the energy term *E*_HB_ calculated by FIRST (Dahiyat et al., 1997). Hydrophobic interactions between carbon or sulfur atoms were taken into account if the distance between these atoms was less than the sum of their van der Waals radii (C: 1.7 Å; S: 1.8 Å) plus an offset of *D*_cut_ = 0.25 Å (Rader et al., 2002).

To elucidate the hierarchy of structural stability in a biomolecule, a trajectory of network states {*σ*}, generated by successively removing hydrogen bond constraints in the order of increasing strength (Hespenheide et al., 2002; Rader et al., 2002; Radestock and Gohlke, 2008; Rader, 2009), was analyzed. In this process, only those hydrogen bonds are retained in a network state *σ* that have an energy *E*_HB_ ≤ *E*_cut_. Altered biomolecular stability along a constraint dilution trajectory was quantified based on neighbor stability maps (*rc*_*ij*, neighbor_ with *i*, *j* being residue numbers) (Rathi et al., 2015).

$$rc_{ij}=\min\left\{E_{cut}|\exists c\in C^{E_{cut}}:R_{i}\wedge R_{j}\in c\right\}$$

Here, only short-range rigid contacts were considered that have ≥ 1 pair of heavy atoms of the residue pair *R*_{*i*,*j*}_ separated by a distance ≤ 4.5 Å (Skolnick et al., 1997). A rigid contact *rc*_*ij*_ between pairs of residues ceases to exist when both residues stop sharing the same rigid cluster *c* of a set of rigid clusters *C* ^*E*cut^. The double sum

$$E_{\text{CNA}}=\sum_{i}^{n}\sum_{j>1}^{n}rc_{ij,\text{neighbor}}$$ (4)

yields the chemical potential energy (*E*_CNA_) due to non-covalent bonding, obtained from the coarse-grained, residue-wise network representation of the underlying biomolecular structure (Rathi et al., 2015; Pfleger et al., 2017). A per-residue decomposition of this equation yields the chemical potential energy of residue *i* because of the *n* short-range rigid contacts the residue is involved in:

$$E_{i,\text{CNA}}=\frac{1}{2}\sum_{j\neq i}^{n}rc_{ij,\text{neighbor}}$$ (5)

We extracted the conformational ensembles that served as input to CNA as 1,000 snapshots from the 200–300 ns time interval of the MD simulations described in section “2.6.2 MD simulations”.

#### 2.6.4 Postprocessing and Data Analysis

Postprocessing and analysis of the MD trajectories were performed with CPPTRAJ (Roe and Cheatham, 2013) as implemented in AmberTools20 (Case et al., 2020). Unless stated otherwise, averages for observables from the MD simulations are expressed as grand mean ± standard error (SEM), calculated from the time averages over the four subunits, which were then averaged over the *n* = 5 trajectories. Error propagation was performed using the uncertainties python package (Lebigot, 2021).

## 3 Results

### 3.1 Effects of Additional Amino Acid Sequences (AAASs) Between Channel Gate and C-Linker on Steady-State Activation

Figure 1A shows the comparative model of an HCN2 channel, based on the templates of hHCN1 and hHCN4 channels. HCN channels are primarily activated by hyperpolarizing voltages, while binding of cAMP enhances opening by accelerating activation, decelerating deactivation, and increasing the current amplitude (Figure 1B). Consequently, steady-state activation is shifted to less negative voltages (black curves in Figure 1C). To study how the effect of cAMP is transmitted from the cyclic nucleotide-binding site to the pore domain, we progressively uncoupled the C-linker from the S6-helix by adding one to five glycines between the last residue of the S6-helix, S441, and the first residue of the A′-helix of the C-linker, L442, resulting in the constructs 1G, 2G, 3G, 4G, and 5G (Figure 1C).

All five constructs formed functional channels in *Xenopus laevis* oocytes. Voltage families ranging from −70 to −150 mV with 10 mV increments were applied to analyze steady-state activation by fitting the Boltzman equation (Eq. Error! Digit expected.) to normalized current amplitudes (see Materials and Methods), yielding the voltage of half-maximum current, *V*_1/2_ (Table 2), and the effective gating charge, *zδ* (Figures 1C–E).

**TABLE 2:** Half-maximum activation in the absence of cAMP and at 10 µM cAMP (mean ± SEM). Number of recordings, *n,* are given in brackets for each construct and condition.

Construct	*V*_1/2_ (mV) w/o cAMP	*V*_1/2_ (mV) at 10 µM cAMP
HCN2	−119.6 ± 1.8 (*n* = 19)	−100.6 ± 1.5 (*n* = 17)
1G	−110.6 ± 1.8 (*n* = 12)	−106.6 ± 1.7 (*n* = 9)
2G	−112.3 ± 1.5 (*n* = 17)	−108.0 ± 1.9 (*n* = 18)
3G	−119.4 ± 1.6 (*n* = 5)	−119.1 ± 1.3 (*n* = 5)
4G	−98.5 ± 2.4 (*n* = 4)	−94.7 ± 1.7 (*n* = 6)
5G	−123.7 ± 2.4 (*n* = 6)	−120.1 ± 1.7 (*n* = 8)

The mean *V*_1/2_ for HCN2 was −119.6 ± 1.8 mV, matching earlier results from our lab (Sunkara et al., 2018). For 1G and 2G, steady-state activation in the absence of cAMP was shifted to more depolarized voltages, resulting in *V*_1/2_ = −110.6 ± 1.8 mV and −112.3 ± 1.5 mV, respectively. These values are significantly different from HCN2 but not from each other. As expected, adding a saturating concentration of 10 µM cAMP to HCN2 resulted in a shift of *V*_1/2_ to more depolarized voltages (*V*_1/2_ = −100.9 ± 1.5 mV). In contrast, adding 10 µM cAMP to 1G or 2G did not affect steady-state activation (Figure 1D). It is noteworthy that 2G showed an additional effect compared to HCN2: The currents regularly decayed after passing a maximum, resembling the inactivation process in spHCN channels in the absence of cAMP (Supplementary Figure S1) (Shin et al., 2004; Dai et al., 2021). Because the late current at the end of the pulse and, thus, the tail current used for forming the Boltzmann analysis were compromised, we corrected the currents for this decay in the analysis (Figure 1). A detailed description of the correction procedure is described in the Supplementary Materials. We are aware that the two processes of inactivation and activation cannot be fully separated using this approach. Consequently, the correction will not reveal a fully uncompromised activation. Therefore, we do not interpret the 2G results extensively herein.

For 3G and 5G, *V*_1/2_ values in the absence of cAMP were similar to those from HCN2 with *V*_1/2_ = −119.4 ± 1.6 mV for 3G and −123.7 ± 2.4 for 5G. As in 1G and 2G, 10 µM cAMP had no significant effect on *V*_1/2_ (3G: *V*_1/2_ = −119.1 ± 1.3 mV; 5G: *V*_1/2_ = −120.1 ± 1.7 mV).

Notably, at zero cAMP, 4G showed the most pronounced difference to HCN2. With *V*_1/2_ = −98.5 ± 2.4 mV, it resembled HCN2 in the presence of saturating cAMP (−100.9 ± 1.5 mV). As in the other insertion constructs, adding cAMP to 4G had no effect (*V*_1/2_ = −94.7 ± 1.7 mV).

To analyze the effects of the glycine insertions on voltage sensitivity, we compared *zδ* values obtained from the Boltzmann fit, specifying the equivalent gating charges moving through the electric field across the membrane. The data variability for 1G with cAMP and 2G with and without cAMP was higher than for all other cases due to a considerable number of recordings giving exceptionally high *zδ* values. For 2G, we assume that the reason for this is the unusual gating behavior described above, resulting in erroneously steep Boltzmann relationships. Such a gating behavior also seems to appear in 1G with cAMP, but to a lesser extent, so that the current decay observed in 1G was not as visible as for 2G (Figure 1). Moreover, for 3G and 4G with and without cAMP, the *zδ* values were lower than the respective HCN2 values (Figure 1).

Together, these data suggest that the function of HCN2 is more disturbed in 4G than in the other four constructs, apparently by a stronger uncoupling of the effects of cAMP and voltage on activation.

### 3.2 Effects of AAASs Between Channel Gate and C-Linker on Structural Dynamics and Statics

**FIGURE 2:**
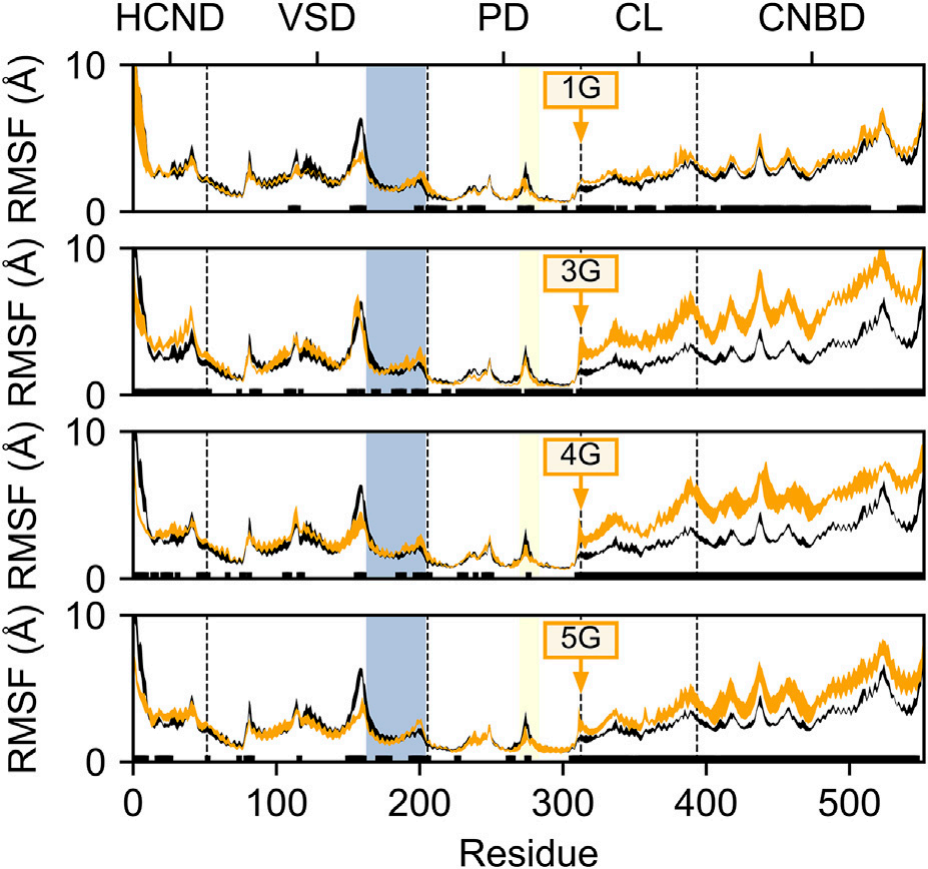
Residue mobility in wildtype HCN2 and the 1G, 3G, 4G, and 5G constructs. Residue-wise root-mean-square fluctuation (RMSF) in MD simulations of HCN2 (black line) and 1G, 3G, 4G, and 5G (orange lines, from top to bottom). The thickness of the lines represents the standard error across all subunits (*n* = 4). Residues with significant differences (*p* < 0.05) between HCN2 and the respective construct are indicated with a black bar above the *x*-axis. Residue numbers are according to the AMBER numbering; domains are denoted above the plots; domain boundaries are indicated by a vertical dashed line. The S4-helix in the VSD is marked with a blue background, and the glycine insertion site is indicated with an orange arrow. For consistency, RMSF values for the glycine insertions were omitted from each subplot.

To determine whether and how the glycine insertions between the S6 and A′-helices affect the structural dynamics of HCN2, we first studied the differences between the domain-wise mobility in the 1G to 5G variants and the wildtype channel. To this end, we determined the residue-wise root mean-square fluctuations (RMSF) after the structural superposition of all MD snapshots onto the pore domain, which is the structurally most invariant region in HCN2. The mobility of the CL and CNBD domains is increased in the 1G to 5G variants compared to wildtype channels, whereas the mobility of the HCND and VSD domains hardly differed from that of wildtype channels (Figure 2). Interestingly, the increase in mobility in CL and CNBD did not correlate with the number of inserted glycine residues. In 1G, the average residue-wise mobility in CL and CNBD increased by 0.42 ± 0.02 Å (Figure 2A). The most pronounced increases occurred in 3G (2.90 ± 0.03 Å, Figure 2B) and 4G (2.44 ± 0.03 Å, Figure 2C), but mobility decreased again in 5G (1.50 ± 0.03 Å, Figure 2D).

**FIGURE 3:**
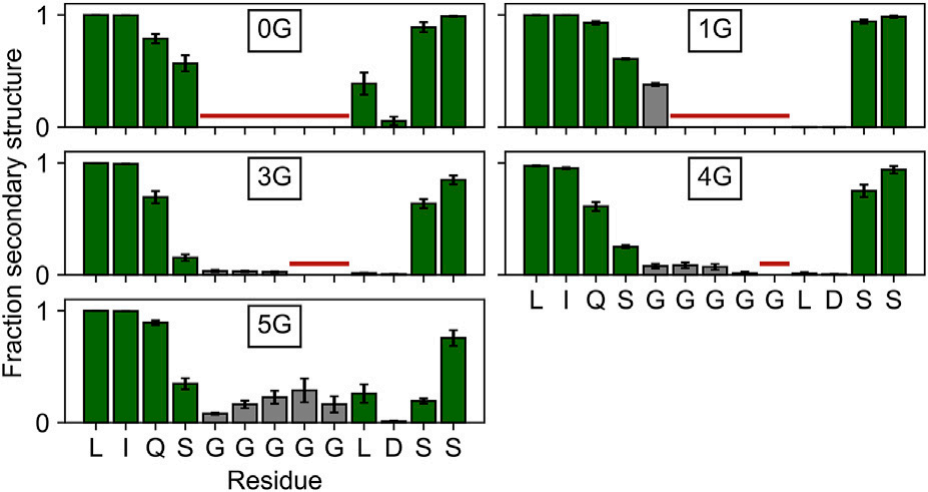
Secondary structure content in and around the insertion site. The secondary structure content of the four residues adjacent to the insertion site is depicted with green bars, that of the glycine insertions with gray bars. Residues not present in the wildtype or a particular construct are marked with a horizontal red line. Any β-strand or helix–but no turns or bends–were counted as secondary structure elements.

Since the lower mobility in 5G suggests secondary structure formation, we inspected the average secondary structure content within the region of the insertion. In 5G, the secondary structure content (18.5 ± 5.8%) is significantly (*p* < 0.05) increased within the glycine insertion when compared to the 3G (3.1 ± 0.8%) and 4G (6.3 ± 2.1%) constructs (Figure 3), but not when compared to the 1G construct (38.0 ± 2.7%). 3_10_ helices, β-sheets, and stable turns are the dominant secondary structure elements in the 5G insertion (Supplementary Movie S1). Note that a PG_II_ (polyglycine type II) secondary structure would form only in the presence of neighboring polyGly chains (Crick and Rich, 1955). Since no structure has been deposited in the PDB in which two α-helices are joined by five glycine residues, a comparison with other structures to substantiate this secondary structure formation is not possible.

**FIGURE 4:**
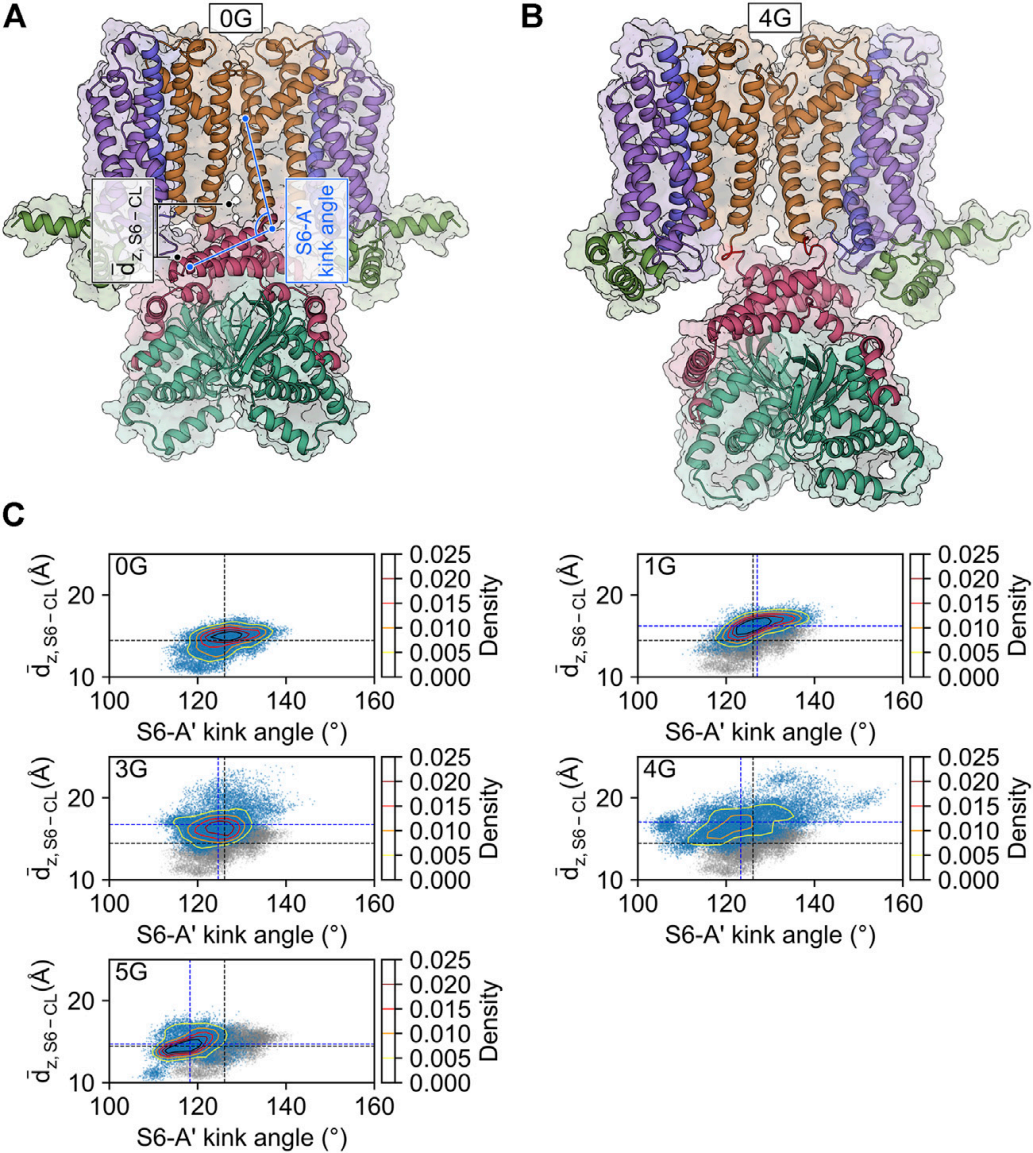
CL-CNBD displacement in glycine linker-carrying constructs. **(A)** Starting structure for the MD simulation of the HCN2 wildtype channel (0G). The color scheme corresponds to the one in Figure 1A. The geometric parameters used to characterize the conformational changes are the vertical distance between the C-terminus of the S6-helix and the C-linker of the counter-clockwise preceding subunit as viewed from the extracellular side (*d*_z, S6 – CL_, black), and the S6-A′ kink angle (light blue). **(B)** Snapshot of the final state of the second simulation of 4G. The color scheme is identical to that in panel A, with the glycine linkers colored in red. Compared to the wild type, 4G displays a pronounced downward displacement of the CL-CNBD. **(C)** Scatter and contour plots of the two geometric parameters in the MD simulations of 0G, 1G, 3G, 4G, and 5G. Values of the average structure of HCN2 are indicated with dashed black lines and those of the constructs with dashed blue lines. In the plots for the glycine linker constructs, the scatter plot for HCN2 is shown in gray for comparison.

The increased mobility of the CL-CNBD relative to the other structural elements suggests that the CL-CNBD motions may be uncoupled from motions of the other domains in the glycine variants. These data may explain why cAMP does not affect the activation of 1G to 5G in the electrophysiological experiments (Figures 1C, D). To substantiate this, we studied the conformational changes that accompany increased mobility. Upon visual inspection of the MD trajectories, we observed vertical movements of the CL-CNBD with respect to the pore domain. Particularly for 3G and 4G, this movement is reflected by an increase in the distance between the z-coordinates of the centers of mass of the four C-terminal residues of the S6 helices and the z-coordinates of the centers of mass of the C-linker of the counter-clockwise preceding subunit as viewed from the extracellular side, indicating a downward displacement of the CL-CNBD (Figure 4A). The downward displacement (Figure 4B) was typically concomitant with an increase in the kink angle between the S6 and A′-helix (Figures 4A,C). Therefore, this conformational change is likely a direct consequence of the high flexibility of the preceding glycine insertions, which turn the junction between the S6 and A′-helix into a flexible hinge within the subunit. While the average distance in the MD ensemble of the wildtype amounted to 14.45 ± 0.01 Å, it increased by 1.77 ± 0.01 Å, 2.30 ± 0.01 Å, and 2.61 ± 0.01 Å in the 1G, 3G, and 4G constructs, respectively.

**FIGURE 5:**
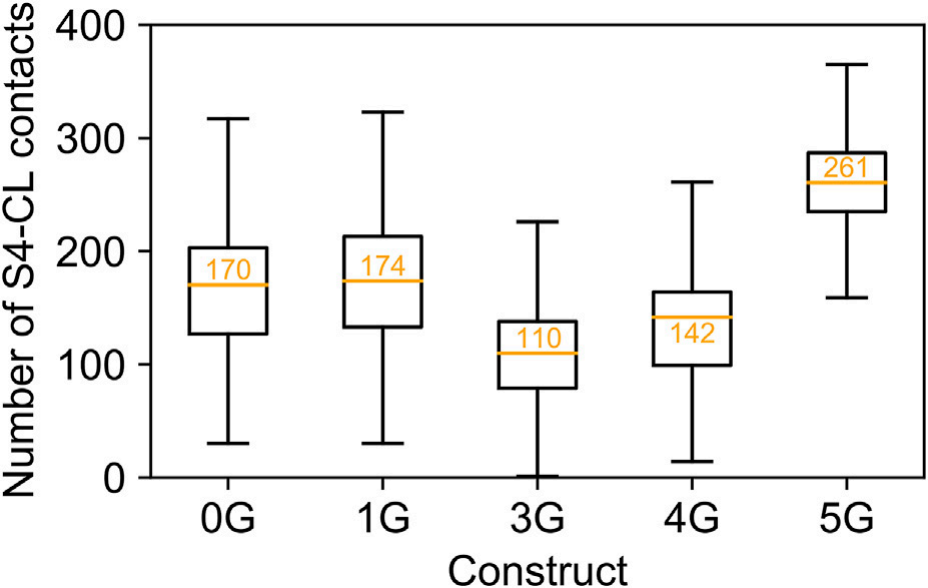
Contacts between S4 and C-linker. Number of contacts between S4 and C-linker in MD simulations of each construct. The values are displayed as a standard boxplot, with the difference that the orange line indicates the mean (additionally depicted as orange number).

Counterintuitively, but consistent with the observed secondary structure formation (Figure 3), the insertion of five glycine residues in 5G led to similar distances like in the wildtype channel (14.70 ± 0.01 Å). The peak values for the downward displacements observed in the simulation ensembles of the 1G-5G constructs with respect to the average distance in the wildtype channel were 5.08 Å for 1G, 9.52 Å for 3G, 10.46 Å for 4G, and 4.83 Å for 5G. These values highlight that the observed downward displacement cannot be a mere consequence of the elongation of the peptide chain. We speculate that the loss of contacts between S4 and CL (Figure 5) resulting from the downward displacement might foster a subsequent downward movement of S4 similar to the one induced by hyperpolarization but smaller in magnitude.

However, given that HCN channels take hundreds of milliseconds to open, we cannot expect to routinely observe such a conformational change of the S4-helix in our simulations, even if activation was accelerated by a factor of ∼25 at weakest hyperpolarization to ∼4 at strongest hyperpolarization (see below and Figure 7). Observation of voltage sensor motion in unbiased MD simulations has so far only been achieved under strongly hyperpolarizing conditions (–550 mV) and simulation times of >20 μs (Kasimova et al., 2019). The reduced equivalent gating charges in 3G and 4G (Figure 1E) support this model in that fewer charges would cross the membrane upon activation if an S4 displacement towards intracellular had already occurred in the closed state.

Although the increase in mobility and the conformational changes of the CL-CNBD relative to the core are very similar in 3G and 4G, *V*_1/2_ is increased only in 4G but is similar to HCN2 in 3G (Figure 1D). We suspected that structural stabilization of certain channel regions in 3G counterbalances the priming of S4 in this construct. Therefore, we sought to identify regions in the constructs that are structurally stabilized or destabilized compared to HCN2 using the rigidity theory-based CNA (Pfleger et al., 2013b). In CNA, biomolecules are represented as constraint networks and then decomposed into rigid clusters and flexible hinge regions according to rigidity theory (Jacobs and Thorpe, 1995; Hermans et al., 2017). Post-processing of constraint dilution simulations as implemented in CNA (Radestock and Gohlke, 2011; Rathi et al., 2015) provides information on the contributions of individual residues to local stability (Pfleger et al., 2013a). Comparing these contributions between wild-type and a construct can thus reveal which regions in the construct become more flexible or rigid. Note that these static analyses are time-independent (Rathi et al., 2015) and, hence, may detect structural (de)stabilizations before these lead to conformational changes.

**FIGURE 6:**
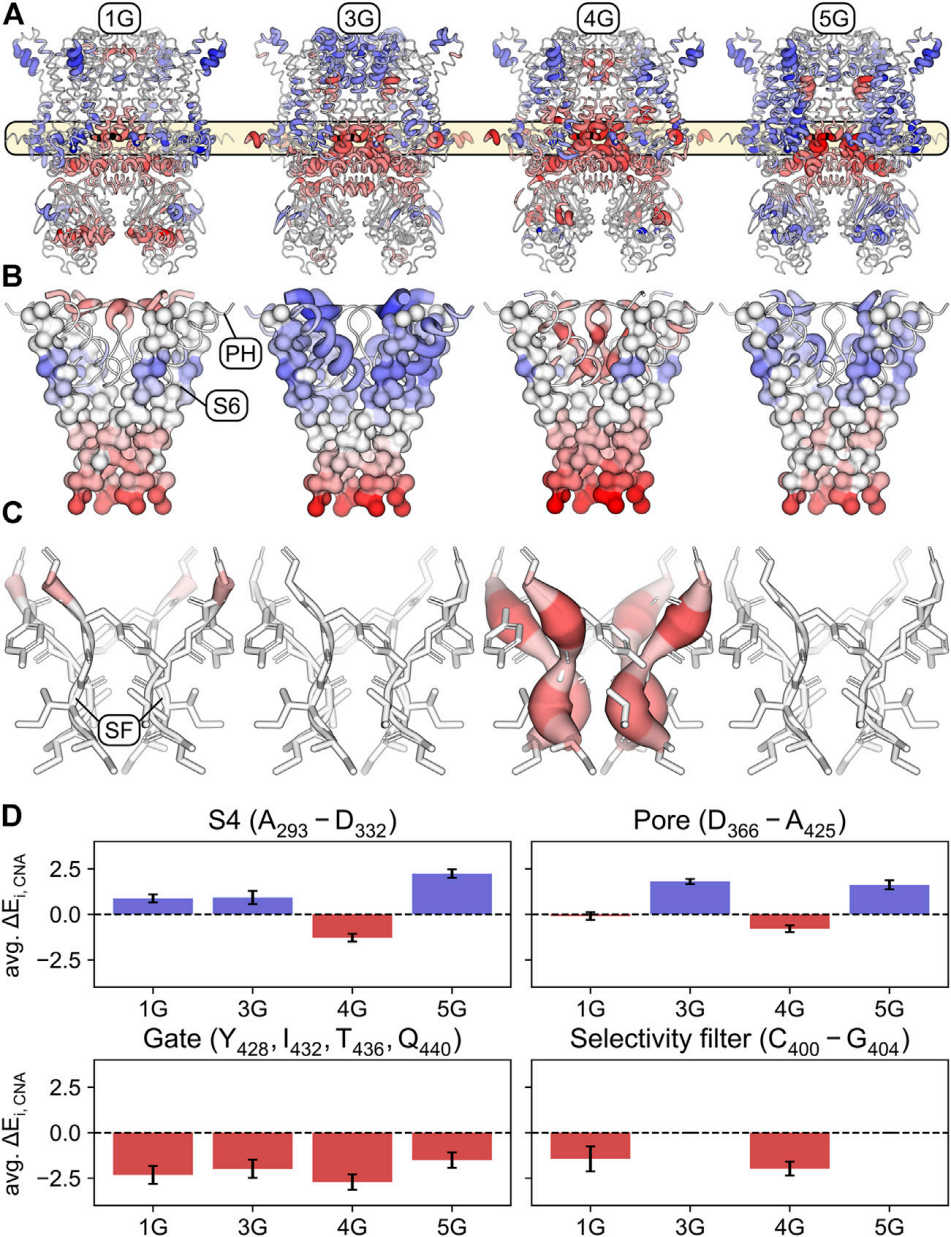
Stabilized and destabilized regions of the glycine-linker constructs according to rigidity analyses. **(A)** Mapping of the differences Δ*E*_*i*,CNA_ = *E*_*i*,CNA(0G)_ − *E*_*i*,CNA(*n*G)_ – averaged across simulations and subunits – onto the structure of the wildtype channel (0G). Regions that are significantly stabilized in the respective construct compared to 0G (positive Δ*E*_*i*,CNA_ values) are shown in shades of blue. Regions that are significantly destabilized compared to 0G (negative Δ*E*_*i*,CNA_ values) are shown in shades of red. Shading is defined by a divergent color map with Δ*E*_*i*,CNA_ ≤ -5.5 kcal mol^−1^: red, Δ*E*_*i*,CNA_ = 0 kcal mol^−1^: white, and Δ*E*_*i*,CNA_ ≥ 5.5 kcal mol^−1^: blue. The thickness of the cartoon representation is scaled using the absolute value |Δ*E*_*i*,CNA_|. Residues for which the differences were not significant (*p* ≥ 0.05) were assigned Δ*E*_*i*,CNA_ = 0 kcal mol^−1^. **(B)** Similar representation as in panel A with a focus on S6, the pore helix, and the selectivity filter. S6 is depicted in a surface representation of its C-alpha trace. **(C)** Same representation as in panel A with a focus on the selectivity filter. **(D)** Average Δ*E*_*i*,CNA_ over the S4 (top left), the upper part of the pore (top right), the S6 gate (bottom left), and the selectivity filter (bottom right). Error bars indicate standard deviations.

Expectedly, and consistent with the increased mobility of the domains following the insertion (Figure 2), the structural flexibility is markedly increased in the regions preceding and following the insertion sites (i.e., the C-terminal end of S6 and the A′ and B’ helices in the C-linker) in all constructs compared to the wildtype channel (Figure 6A). This supports our suggestion that the motions of the CL-CNBD and the other domains is decoupled in the constructs, thereby abolishing the cAMP effect. As a consequence, the gate is destabilized in all constructs (Figure 6D). Moreover, parts of the selectivity filter in 1G and the entire selectivity filter in 4G are destabilized, in contrast to 3G and 5G (Figures 6C, D). Additionally, parts of the S4 are destabilized in 4G, but stabilized in all other constructs (Figure 6D). Most notably, the stability of the upper pore region is increased in 3G and decreased in 4G (Figures 6B, D). Our data suggest that destabilization of the upper pore region is determined by the extent and strength of the destabilization of the S6 C-terminus. In 1G and 4G, destabilization spreads furthest towards the pore domain (Figure 6B), but is more pronounced in 4G and propagates to the selectivity filter. However, in 3G and 5G, the destabilization spreads less towards the pore domain (Figure 6B), which might not suffice to destabilize the selectivity filter similarly. Taken together, these data suggest that destabilization of the S6 C-terminus in 1G and 4G causes destabilization of the selectivity filter and, in 4G, additionally destabilizes other parts of the upper pore region. This could facilitate the passage of ions through the selectivity filter or pore and thus explain the increased *V*_1/2_ values in these constructs.

### 3.3 Increased Flexibility Causes Fast Activation Kinetics

The observed higher flexibility of the CL-CNBD domains and of the gate structure (here defined by amino acids Y428, I432, T436, and Q440) led us to hypothesize that these changes cause an accelerated activation when skipping to hyperpolarizing voltage. The additionally destabilized S4 domain and selectivity filter in 4G should further accelerate activation compared to the other insertion constructs. To test this hypothesis, we used the patch-clamp technique to analyze time courses of activation for all five constructs at different voltages and compared the results with those from HCN2 wildtype in the absence and presence of cAMP.

**FIGURE 7:**
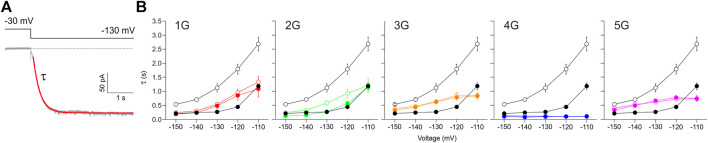
Effect of inserted glycines on activation kinetics. **(A)** Example trace for 1G illustrating a fitted exponential curve (red) to obtain the time constant of activation, *τ*, by using Eq. Error! Digit expected **(B)** Plots of activation time constants versus voltage for all five mutant constructs in comparison to HCN2 (black lines and symbols in all five panels). Empty symbols represent cAMP-free condition, filled symbols represent recordings at 10 µM cAMP. Glycine-induced uncoupling interrupts the reciprocal feedback from VSD to CNBD.

The speed of activation was quantified by fitting a single exponential (Eq. Error! Digit expected.) to current time courses, yielding the activation time constant τ (Figure 7A). For HCN2 in the absence of cAMP, *τ* shows the characteristically steep decrease towards hyperpolarizing voltages (Figure 7B). 10 µM cAMP caused the typical decrease of *τ* at all voltages. For all mutant constructs, this accelerating effect of cAMP has vanished in parallel with the lost effect on steady-state activation. For 1G, 2G, 3G, and 5G, the time constants of all constructs were almost always between those of HCN2 without and with cAMP, but with less pronounced voltage dependencies. The data for 2G are largely consistent with the data series for the other constructs, but the exponential fits might be compromised due to the correction procedure. In contrast, 4G showed exceptionally fast activation kinetics, even faster than HCN2 at saturated cAMP. Moreover, in 4G, the voltage dependence of activation has completely vanished (Figure 7B). Hence, these experimental data match the predictions of the MD simulations above.

Previous data from our group showed that there is reciprocity between ligand binding and channel activation in HCN2: By measuring ligand binding and channel activation in parallel, using confocal patch-clamp fluorometry (cPCF) with a fluorescent cAMP derivative, we experimentally verified this context by showing an activation-induced increase of the binding affinity (Kusch et al., 2010).

In line with this, we expected that channel constructs that are not reactive to ligand binding do not show an activation-induced affinity increase. For 1G and 4G, we tested this by recording ligand binding in parallel with channel activation using cPCF. Similar to previous measurements, ligand binding was quantified by the fluorescence intensity of a fluorescent cAMP derivative binding to the channels in the excised patch membrane (Biskup et al., 2007; Kusch et al., 2010). As cAMP derivative, 8-AHT-Cy3B-cAMP (f1cAMP) was used (Otte et al., 2019; Pfleger et al., 2021), which contains a different dye moiety and a longer linker compared to the originally used cAMP derivative 8-AET-Dy547-cAMP (fcAMP) (Kusch et al., 2010) (see also Materials and Methods).

**FIGURE 8:**
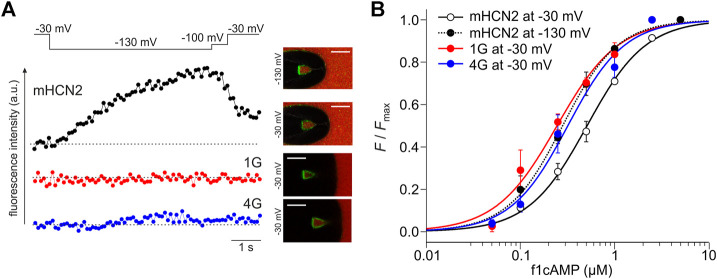
Reciprocity between binding and gating in glycine constructs. **(A)** Examples of fluorescence traces for HCN2, 1G, and 4G at 0.25 µM f1cAMP. The confocal fluorescence images in the insets show pipette tips holding membrane patches with the given constructs expressed. Green fluorescence arises from f1cAMP binding to the channel. **(B)** Concentration-binding curves for HCN2 at −30 and −130 mV, 1G at −30 mV, and 4G at −30 mV. Color coded lines were obtained by fitting the Hill equation (Eq. Error! Digit expected.) to the data points (dotted line for mHCN2 at −130 mV, solid lines for 1G, 4G and mHCN at −30 mV). Single data points are averages of recordings from 3 to 20 different patches (except mHCN2 at 1 µM: *n* = 1).

In line with our previous results, in HCN2, the fluorescence increased when activating the channels by stepping from −30 to −130 mV (Figure 8). The channels transit from a low-affinity state to a high-affinity state. Neither for 1G nor 4G did we observe such an increase in cAMP binding upon activation. Figure 8A shows representative fluorescence traces obtained at 0.25 µM f1cAMP.

To understand at which affinity level the insertion constructs are stabilized, we measured concentration-binding relationships for 1G and 4G and compared them with HCN2 (Figure 8B). To this end, the fluorescence intensity *F*, measured at different f1cAMP concentrations at either −30 mV or −130 mV, was related to the maximum fluorescence intensity, *F*_max_, measured at a saturating concentration of 2.5 µM f1cAMP and −130 mV. *F*/*F*_max_ values are averages from 3 to 9 recordings. Fitting the Hill equation for ligand binding (Eq. Error! Digit expected.) to the averaged data yielded the concentration of half-maximum binding, *BC*_50_, and the Hill coefficient of binding, *H*_b_ (Figure 8B).

The binding affinity *BC*_50_ of HCN2 not activated by voltage was 0.51 µM. Notably, the binding affinity of both 1G (*BC*_50_ = 0.24 µM) and 4G (*BC*_50_ = 0.32 µM) not activated by voltage approximates the binding affinity of HCN2 activated by voltage (*BC*_50_ = 0.28 µM). These data suggest that both insertion constructs exist already in the high-affinity state when not being activated by voltage, a state that HCN2 can adopt only at activation by voltage. Therefore, in both 1G and 4G, a further increase of binding affinity is absent. The Hill coefficient was similar for all constructs under all conditions (HCN2 at −30 mV: *H*_b_ = 1.4; HCN2 at −130 mV: *H*_b_ = 1.5; 1G: *H*_b_ = 1.3; 4G: *H*_b_ = 1.4).

## 4 Discussion

The aim of this study was to further analyze the processes underlying cAMP modulation of HCN channels. We manipulated the interaction between the CL-CNBD and membrane portion by inserting one through five glycines directly after the activation gate formed by the S6-helix bundle, yielding the constructs 1G-5G. We then studied the effects of these insertions on structure and function using molecular simulation approaches and electrophysiological techniques.

All five insertion constructs led to channels that could be activated by hyperpolarizing voltage. Voltage-dependent activation at steady-state and in the absence of cAMP in 1G and 2G was shifted moderately towards depolarizing voltages (Figure 1). Thus, in these constructs, channel opening was favored moderately compared to HCN2. Regarding that in HCN2 the empty CNBD exerts an autoinhibitory effect on channel gating (Wainger et al., 2001), the observed *V*_1/2_ shift can be interpreted as a reduction of such an effect. In empty 4G channels, activation was shifted more to depolarizing voltages, resembling *V*_1/2_ values of cAMP-saturated HCN2. By contrast, in 3G and 5G, steady-state activation was not affected (Figure 1). These data indicate that the extent of the functional effects of AAA insertion is not simply a function of the length of such a sequence but that each AAA sequence exerts specific effects. Steady-state activation also showed that in all five constructs the characteristic cAMP-induced shift of *V*_1/2_ to more depolarized voltages was completely abolished (Figure 1), indicating that insertion of only one glycine already suffices to uncouple the CL-CNBD portion from the membrane portion.

To gain deeper insight into the structural and functional changes of the AAAs insertion, we performed different computational approaches. First, we studied the domain-wise mobility in all five constructs and compared it to the mobility in HCN2 (Figure 2). For all insertions, the mobility is increased in the CL-CNBD domain, but hardly in the HCN domain and VSD. Interestingly, the highest mobility was found for 3G and 4G. The reason why a chain of 5 glycines did not cause higher mobility than a chain of 4 glycines is presumably due to secondary structure formation in 5G (Figure 3). Nevertheless, independent of the degree of the mobility increase, these changes in the insertion constructs are suggested to be a consequence of a CL-CNBD uncoupled from the other channel domains. So far, these data show that the insertion of the additional amino acids is a suitable tool to uncouple two protein regions from each other to understand their interaction better.

What are the structural consequences for the channel conformation near the insertion site? The glycine residues were inserted directly after the S6 segment and the beginning of the A′-helix in the C-linker. The MD simulations revealed a downward displacement of the CL-CNBD. This downward displacement is accompanied by an increase in the kink angle between S6 and A′-helix, which is most pronounced in 3G and 4G (Figure 4). Furthermore, the junction between S6 and A′-helix is turned into a flexible hinge. Both S6 and A′-helix play a relevant role in the opening process: The C-terminal ends of S6 form the channel gate, a right-handed helical bundle (Rothberg et al., 2002), which is held closed by the presence of the S4-helix at depolarized voltages (Lee and MacKinnon, 2017). The C-linkers of the four subunits tetramerize to form a disc-like gating ring. The characteristically long S4-helix allows the S4-S5 linker to directly contact the C-linker of the left neighboring subunit (viewed from extracellular). At hyperpolarized voltages, the displacement of S4 releases the constraints on the C-linker and on S6, which is followed by an unwinding of the gate due to a rotational movement of the gating ring (Lee and MacKinnon, 2017). Interestingly, this mechanism is still functional after AAA insertion between the S6 and A′-helix. All insertion constructs responded to hyperpolarizing voltages. For 1G, 4G, and eventually also for 2G, the opening was even favored compared to HCN2 wildtype. Based on the gating scenario described above, this could be a consequence of a downward displacement of the S4 helix, favored by a void between S4 and CL that would form upon downward displacement of the CL-CNBD (Figure 5). Disruption of such contacts in response to hyperpolarization has been proposed to energetically favor channel opening (Decher et al., 2004). This presumed downward displacement of the S4-helix is likely similar to the one induced by hyperpolarizing voltages (Männikkö et al., 2002; Bell et al., 2004; Vemana et al., 2004; Vemana et al., 2004; Bruening-Wright and Larsson, 2007; Dai et al., 2019) but to a lesser extent, reducing the energy needed for activation by voltage. The similarity between conformational changes induced by hyperpolarization and conformational changes induced by glycine insertion led us to speculate, that those changes might be responsible for the increased affinity in glycine constructs (see Figure 8).

But why did 3G behave so differently from 4G, despite showing similar downward displacements of the CL-CNBD? To answer this question, we performed a rigidity theory-based Constraint Network Analysis (CNA) (Pfleger et al., 2013b) to identify a structural stabilization of certain channel regions in 3G counterbalancing the priming of S4 in this construct (Figure 6). For all insertion constructs, also the CNA showed markedly increased structural flexibility in the regions preceding and following the inserted AAAs. This supports the conclusion drawn from the mobility analysis that the CL-CNBD is decoupled in the insertion constructs. As a consequence, the gate is destabilized in all constructs. However, a destabilization of the whole selectivity filter and parts of the S4 domain were found exclusively for 4G. The most notable difference between 3G and 4G is the increased stability of the upper pore region (defined as amino acids D366-A425) in 3G and decreased stability of the same region in 4G.

How can we explain the missing cAMP-effect in the insertion constructs? Previous publications showed that the binding of cAMP to the CNBD induces an iris-like rotation of the gating ring, which is in the direction of pore opening (counter-clockwise viewed from the extracellular side). This unwraps the right-handed S6 bundle, but to a lesser extent than the unwrapping caused by hyperpolarization. Therefore, cAMP binding supports channel opening but is not able to induce it without hyperpolarization (Shin et al., 2004; Lee and MacKinnon, 2017; Weissgraeber et al., 2017; Gross et al., 2018; Marchesi et al., 2018). Herein, cAMP did not show an effect on the insertion constructs, indicating that the conformational changes needed for priming the CL-disk rotation or the transmission of this rotation to the gate are compromised. Our results from MD analysis support both options: 1) The higher flexibility seen in the CL-CNBD portions of all constructs might affect the binding-induced conformational changes in the CNBD and their upwards transmission along the C-linker; 2) The high flexibility of the hinge and the increased angle between A′-helix and S6 might hamper the transition of the CL rotation to the S6-helix bundle.

Based on the mentioned gating scenario, an accelerated channel activation is expected from the higher flexibility of the S4 domain and S6 gate region and a downward displacement of the CL-CNBD in the sense of priming. We tested this by measuring current responses to different hyperpolarizing voltage jumps and determining the activation time constant τ, both in the absence and presence of cAMP. As expected, for all insertion constructs, the activation speed in the absence of cAMP was faster compared to HCN2. The characteristic accelerating effect of cAMP vanished in all cases. One potential reason for such an acceleration of activation kinetics may be a depolarizing shift in the voltage dependence. However, if this was the only reason for the accelerations observed herein, the respective τ_act_-voltage curves would show a similar slope as the wildtype curve, just covering a different voltage range. For all five glycine constructs, the slopes were clearly different from HCN2, indicating that the depolarizing shift in voltage dependence cannot account for the speed of activation alone. Interestingly, again, 4G showed an exceptional behavior: the activation was fastest among all insertion constructs and even faster as seen for cAMP-saturated HCN2. This matches the exceptionally high structural flexibility of the 4G S4 and the destabilization of the whole selectivity filter, which both may facilitate conformational changes, allowing for fast channel opening.

Regarding the impact of cAMP on the channel protein, the data obtained showed that both communication pathways, from the CNBD to the transmembrane part and, vice versa, from the transmembrane part to the CNBD, are corrupted by the AAAs insertion near the gate. In the most simple scenario, this is solely caused by the higher flexibility of S6, A′-helix, and the hinge between them, interrupting the transmission of conformational changes in both directions. However, Porro and co-workers suggested in a recent report a mechanism by which the two stimuli of activation, hyperpolarizing voltage and cAMP binding, are integrated by the HCN domain via forming a mechanical continuum between the voltage sensor and the CNBD (Porro et al., 2019). In this scenario, information between the CNBD and VSD is transmitted at the periphery of the channel, bypassing the pore. Considering these findings, not only the higher flexibility close to the central axis of the channel but also regions and interactions further away are suggested to be significantly affected by the AAAs insertion (Figure 6A).

The combined electrophysiological and computational results provide new insights into the intricate activation of HCN2 channels.

## Data Availability

The datasets presented in this study can be found in online repositories. The names of the repository/repositories and accession number(s) can be found in the article/Supplementary Material.
